# Comparison of different methods in analyzing short-term air pollution effects in a cohort study of susceptible individuals

**DOI:** 10.1186/1742-5573-3-10

**Published:** 2006-08-09

**Authors:** Annette Peters, Stephanie von Klot, Niklas Berglind, Allmut Hörmann, Hannelore Löwel, Fredrik Nyberg, Juha Pekkanen, Carlo A Perucci, Massimo Stafoggia, Jordi Sunyer, Pekka Tiittanen, Francesco Forastiere

**Affiliations:** 1 GSF-National Research Center for Environment and Health, Institute of Epidemiology, Neuherberg, Germany; 2 Institute of Environmental Medicine, Karolinska Institutet, Sweden; 3 Dept. of Occupational and Environmental Health, Stockholm County Council, Sweden; 4 GSF-National Research Center for Environment and Health, Institute of Health Economics and Management, Neuherberg, Germany; 5 AstraZeneca R&D, Mölndal, Sweden; 6 Unit of Environmental Epidemiology, KTL – National Public Health Institute, Kuopio, Finland; 7 Department of Epidemiology, Rome E Health Authority, Rome, Italy; 8 IMIM, Barcelona, Spain

## Abstract

**Background:**

Short-term fluctuations of ambient air pollution have been associated with exacerbation of cardiovascular disease. A multi-city study was designed to assess the probability of recurrent hospitalization in a cohort of incident myocardial infarction survivors in five European cities. The objective of this paper is to discuss the methods for analyzing short-term health effects in a cohort study based on a case-series.

**Methods:**

Three methods were considered for the analyses of the cohort data: Poisson regression approach, case-crossover analyses and extended Cox regression analyses. The major challenge of these analyses is to appropriately consider changes within the cohort over time due to changes in the underlying risk following a myocardial infarction, slow time trends in risk factors within the population, dynamic cohort size and seasonal variation.

**Results:**

Poisson regression analyses, case-crossover analyses and Extended Cox regression analyses gave similar results. Application of smoothing methods showed the capability to adequately model the complex time trends.

**Conclusion:**

From a practical point of view, Poisson regression analyses are less time-consuming, and therefore might be used for confounder selection and most of the analyses. However, replication of the results with Cox models is desirable to assure that the results are independent of the analytical approach used. In addition, extended Cox regression analyses would allow a joint estimation of long-term and short-term health effects of time-varying exposures.

## Background

Ambient air pollution has been associated with increases in acute morbidity and mortality [1]. Patients with underlying chronic diseases such as for example diabetes, ischemic heart disease or heart failure may be at particular risk for the effects of ambient air pollution. Series of incident cases may be followed over time to assess the impact of short-term fluctuation on recurrent disease exacerbation. These studies deviate from the classical cohort as well as time-series designs. The major statistical challenge in these studies is the control for changes within the cohort over time due to changes in the underlying risk following disease inception, slow time trends in risk factors within population, dynamic cohort size and seasonal variation. The impact of time is particular important when effects of air pollution are considered, because concentrations of air pollutants vary by season and yearly averages of pollutants change due to weather, air pollution control measures and changes in sources.

In order to evaluate the impact of air pollution on patients with pre-existing diseases, previous hospitalization might be used to define a potentially susceptible subgroup [2-4]. Poisson regression analyses and case-crossover analyses have been used to estimate the impact of variations in daily average air pollution concentrations on the risk of death in previously hospitalized subgroups of the population [5-8].

A multi-city study was designed to follow cohorts of myocardial infarction (MI) survivors in five European cities: Augsburg, Barcelona, Helsinki, Rome and Stockholm (HEAPSS-Study). For entry into the cohort, incident first myocardial infarction cases were considered. The outcomes considered were re-infarctions and other related re-hospitalization or death. Ambient air pollution was characterized based on existing air monitoring networks. In addition, condensation particle counters were set up in each location to measure the ambient particle number concentrations (PNC) and to retrospectively estimate PNC for the entire study period in each location.

This paper describes and compares different statistical approaches for analyzing short-term air pollution health effects in a cohort with ongoing recruitment during the follow-up. Three different methods to analyze the cohort data were considered: (a) Poisson regression analyses on the calendar-time axis, (b) case-crossover analyses and (c) extended Cox regression analyses. As an example the analyses of the association between NO_2 _and any cardiac readmission of MI survivors are presented.

## Methods

### Data collection

Incident myocardial infarction survivors were recruited in five European cities (Augsburg, Barcelona, Helsinki, Rome, and Stockholm) during 1992 to 2000 as has been described elsewhere [9]. Briefly, data sources were AMI Registries in Augsburg and Barcelona, administrative databases of hospital admissions in Helsinki, Rome and Stockholm. Enrollment was restricted to residents of the above cities, aged 35 years or more (35–74 in Augsburg, 35–79 in Barcelona) who had their first AMI (index AMI) during the recruitment period. Subsequent first cardiac re-hospitalizations of cohort members within the study area were recorded from day 29 after the index AMI until the individual end of follow-up period defined by death, migration out of the study area, or center specific end of the study. Readmissions of interest were those with primary diagnoses of re-infarctions (ICD-9: 410; ICD-10: i21, i22), acute angina pectoris (ICD-9: 411, 413; ICD-10: i20–i22, i24, i25), dysrhythmia (ICD-9: 427; i46.0, I46.9, i47–i49, r00.1, r00.8) and heart failure (ICD-9:428; ICD-10: i50). Vital status and place of residence at the end of the follow-up period were ascertained for all cohort members.

### Statistical analyses

In the study, MI survivors entered the cohort during an extended period. Once the cohort was complete at least one year of follow-up without recruitment was added. As a consequence the following issues had to be considered in the statistical analyses: (a) the number of subjects at risk increases over the recruitment period, (b) the risk of the cohort for a recurrent event on the calendar-time axis varies based on the proportion of recent MI survivors in the entire cohort. In the following we refer to calendar time when we number the dates during follow-up and to cohort-time when we number the person-time followed.

### Poisson regression analyses

Epidemiological studies in air pollution research have developed techniques using Poisson regression analyses on the calendar-time axis [10]. In a time series analysis the daily counts of events are regressed on the daily predictor variables such as trend, season, weather, and air pollution. This design assumes that the events are independent and that the event rate is changes only slowly over time. The event rate λ_t _at the time point *t *is modeled as follows

ln(*λ*_t_) = *α *+ ∑*β*_*i*_*x*_*it*_

With λ_t _= y_t_/N_t _being the number of cases *y *observed at time *t *divided by the number of subjects *N *at risk at time *t*. The model can be rewritten as

ln(y_t_) = ln(N_t_) + *α *+ ∑*β*_*i*_*x*_*it*_

In time-series analyses it is sometimes assumed that the underlying population at risk does not change, and therefore the population at risk is not modeled explicitly in the regression analyses. However, when applied to a cohort the number of subjects at risk can be explicitly modeled as given in the equation above. In the cohort setting, when analyzing the data on the calendar time axis, the trend captures three different underlying reasons for changes in the rate of MI over time: (a) long-term underlying trends in the study base due to life-style changes, changes in health care or aging of the population, (b) seasonal variation and (c) changing composition of the cohort. Therefore, in the cohort setting proper trend control is very important because it is likely that the effect estimates will be biased if the trend is not correctly specified. Recent discussions on modeling in time-series analyses [11,12] have lead to a broader use of different smoothing techniques in these analyses and different approaches have been used to assess the trend over time [13]. We selected three approaches to model the trend: (a) natural splines, (b) penalized splines and (c) locally weighted least square (loess) smoothers.

In a hierarchical approach potential confounders were selected, including long term trend, season, days of the week, holidays and meteorology, before adding air pollution concentration as independent variable. Generalized additive models were used to allow for non-parametric functions of the confounders in R (The R Foundation for Statistical Computing Version 1.8.1) using the package "mgcv" (version 0.9–6) [14-16]. All models included the natural logarithm of the number of persons at risk each day as offset and the daily number of events as outcome variable. To allow for possible over- or under-dispersion the quasi-likelihood family was used to estimate the parameters without specifying the underlying distribution function. Penalized regression splines were tested for the continuous confounder variables. The choice of degrees of freedom was left to the algorithm ("magic") in the "mgcv" – package that minimized the Generalized Cross Validation (GCV) criterion. The default of 10 knots as starting value was adjusted to higher values if necessary due to the structure of the data. If the smooth function was not significant or the estimated degrees of freedom were less than two, a linear term was tested instead. Decisions for keeping a covariate in a model were based on judgment using the p value (<.1), GCV score (the smaller the better), and the autocorrelation function (ACF) (the nearer to zero the better).

Trend was included in the model as an obligatory variable with a penalized spline starting with 6 knots per year to control for long term trends, seasonality and changes in the baseline risk. Then current day temperature and the deviation of the current day temperature from the mean temperature of lag day 1–3 were tested as penalized splines. At least one temperature term had to remain in the model. Thereafter penalized splines of air pressure and relative humidity, and then dummies for days of the week and the city specific indicators (holidays, population decrease) were tested one after the other. Finally the model was "fine tuned" changing the parameters where the decision had not been clear and comparing ACF plots and GCV-score to choose the final covariates to include for the further analyses.

Sensitivity analyses were performed to compare the results of the final models with the estimates obtained when modifying the smoothing-functions used or the confounders entered in the Poisson model. Thus the analyses were repeated using natural cubic splines or loess instead of the penalized splines, with comparable effective degrees of freedom to those in the final model with penalized splines. Temperature was replaced by apparent temperature [17] and dew point temperature. Instead of temperature difference the mean of lag day 1 to 3 was used. The sensitivity of the results to the choice made when selecting the confounders was tested by excluding or including borderline significant confounders that had or had not entered the model. At the same time, those that had been included with a linear term were considered with a smooth function in the sensitivity analyses.

### Case-crossover analyses

Case-crossover analyses have been developed to study transient effects of acute exposures using a case-only design. This design samples information on exposure status from case and referent periods selected from the person-time of the cases. The period of exposure of the cases is selected as a plausible hazard period immediately preceding the event. Referent periods are chosen to represent the exposure distribution in the non-case time periods at risk.

The referent period selection poses the main challenge to the case-crossover analyses. Different sampling designs for referent periods have been proposed to estimate the effects of air pollution. Exposures that do not change in association with the case-status, such as air pollution can be sampled also from person-time after the case occurred [18]. The stratified approach by Lumley and Levy [19] controls time trends by design.

Case-crossover analyses were performed as an alternative to the Poisson regression using conditional logistic regression models in the S-Plus statistical package version 6.0. We used the "coxph" function with a strata statement. The date of each case contributed a hazard period that was matched with referent periods selected with the stratified approach (stratifying criteria were year, month and weekday). It controls for weekday by design. The same confounder variables were included as in the Poisson regression in order to obtain maximum comparability of the models. P-Splines were used as smoothing method for meteorology variables. The effective degrees of freedom of the smoothers in the Poisson models were translated by adjusting the smoothing parameter of the P-splines.

### Proportional hazard models

An alternative possibility is to model the data as cohort data using Cox proportional hazard models. Time t now denotes the time since MI. Extended Cox regression allows for time-varying covariates in survival analyses [20]. The hazard *h *at time *t *is given as

h(t,X(t))=h0(t)exp⁡[∑i=1p1βiXi+∑j=1p2δjXj(t)]
 MathType@MTEF@5@5@+=feaafiart1ev1aaatCvAUfKttLearuWrP9MDH5MBPbIqV92AaeXatLxBI9gBaebbnrfifHhDYfgasaacH8akY=wiFfYdH8Gipec8Eeeu0xXdbba9frFj0=OqFfea0dXdd9vqai=hGuQ8kuc9pgc9s8qqaq=dirpe0xb9q8qiLsFr0=vr0=vr0dc8meaabaqaciaacaGaaeqabaqabeGadaaakeaacqWGObaAcqGGOaakcqWG0baDcqGGSaalcqWGybawcqGGOaakcqWG0baDcqGGPaqkcqGGPaqkcqGH9aqpcqWGObaAdaWgaaWcbaGaeGimaadabeaakiabcIcaOiabdsha0jabcMcaPiGbcwgaLjabcIha4jabcchaWnaadmaabaWaaabCaeaaiiGacqWFYoGydaWgaaWcbaGaemyAaKgabeaakiabdIfaynaaBaaaleaacqWGPbqAaeqaaOGaey4kaSYaaabCaeaacqWF0oazdaWgaaWcbaGaemOAaOgabeaakiabdIfaynaaBaaaleaacqWGQbGAaeqaaOGaeiikaGIaemiDaqNaeiykaKcaleaacqWGQbGAcqGH9aqpcqaIXaqmaeaacqWGWbaCcqaIYaGma0GaeyyeIuoaaSqaaiabdMgaPjabg2da9iabigdaXaqaaiabdchaWjabigdaXaqdcqGHris5aaGccaGLBbGaayzxaaaaaa@631D@

where *X*(*t*) = (*X*_1_, *X*_2_,....., *X*_*p*1_, *X*_1_(*t*), *X*_2_(*t*),....., *X*_*p*2_(*t*)) and *X*_1_, *X*_2_,....., *X*_*p*1 _are time-invariant variables such as for example age or gender, and *X*_1_(*t*), *X*_2_(*t*),....., *X*_*p*2_(*t*) are time-varying variables such as weather or air pollution.

The model makes no assumption about the baseline hazard, and therefore is suitable for the analyses proposed here because the risk for cardiac readmission changes during the follow-up of the MI survivors. While *X*_*j*_(*t*) is varying over time, the hazard model provides only one regression coefficient *δ*_*j *_for each time-varying variable in the model. Thus at time *t*, there is only one value of the variables *X*_*j*_(*t*) that has an effect on the hazard: the value being measured at time *t*. The model therefore assumes a uniform relative risk for all time points and consequently does not per se address the possibility of effect modification of the air pollution effects by length of follow-up.

The association with daily pollution levels was analyzed in SAS statistical software (SAS Institute Inc., Cary, NC, USA, Release 8.02) PROC PHREG using the counting process style of input. For each subject one record for each day at risk was created. All models included the same covariates as the Poisson models, but instead of smooth functions quadratic functions were used. Trend entered as a linear term. As constant covariates age at entry (in years as quadratic function), diabetes, hypertension and sex (as dummy variables) were considered, since they were identified as predictors of survival in a classical Cox regression.

## Results

**Figure 1:**
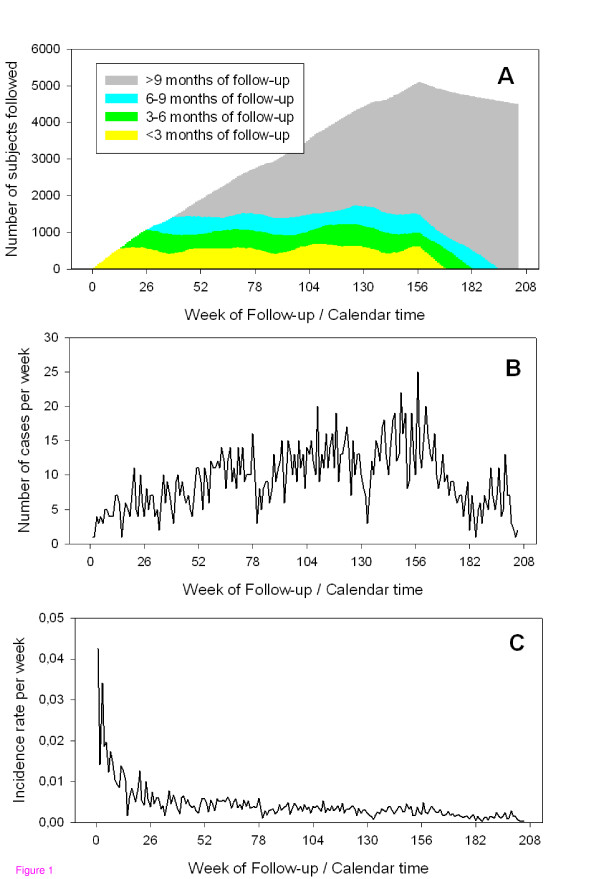
Number of MI survivors followed (a), the number of readmissions to the hospitals due to any cardiac disease during the followup (b) and incidence rate during the follow-up in Rome (c) as part of the HEAPSS study on the calendar-time axis.

**Figure 2:**
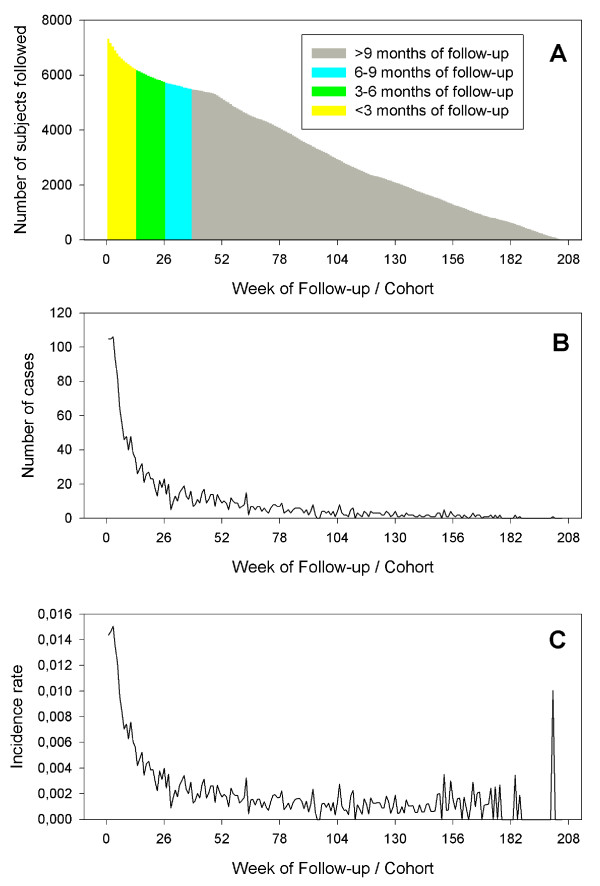
Number of MI survivors followed (a), the number of readmissions to the hospitals due to any cardiac disease during the followup (b) and incidence rate during the follow-up in Rome (c) as part of the HEAPSS study on the cohort time-axis.

Data from Rome is used to illustrate the properties of the data. Rome was selected because it had one of the larger data sets and had clear evidence for seasonal variation. In Rome between 1998 and 2000, 7384 subjects survived at least 28 days after their first MI (Table 1). Between 1998 and 2001, 1916 readmissions for any cardiac disease defined as readmission for angina pectoris, myocardial infarction, congestive heart failure or arrhythmia were observed. Figure 1 describes various aspects of the cohort data for any cardiac readmission of MI survivors in Rome displayed on the calendar time-axis. During the follow-up, the size of the cohort changed daily (figure 1a). In addition, the composition with respect to the distribution of length of follow also changes constantly if considered on the calendar-time axis. Consequently, the number of cases observed at each time-point of the calendar-time axis of the follow-up reflects the size of the cohort and its composition (figure 1b). For each MI survivor, the risk of hospitalization for any cardiac disease is elevated during the first half year of follow-up and thereafter stabilizes (figure 1c). Figure 2 shows the number of subjects followed considering the time of follow-up as time-axis. The number of subjects followed over time steadily decreases due to the occurrence of an event, loss to follow-up or end of the observational period (figure 2a). The number of events (figure 2b) and the incidence rate (figure 2c) observed is greatest at the beginning of the follow-up due to the vulnerability of the patients in the time immediately after the index event. The incidence rate observed after two or three years of follow-up is low (figure 2c).

**Table 1:** Description of the HEAPSS Study population, cardiac readmission as a selected outcome, and NO_2 _concentrations in 5 European cities.

**City**	**Period of Enrollment**	**Period of Follow up**	**Age Range (yrs)**	**Population Size**	**Persons followed**	**Mean Age (yrs)**	**Any cardiac readmission during follow up**		**Mean NO_2 _[μg/m^3^]**	**Maximum NO_2 _[μg/m^3^]**
							**N**	**λ**		
**Augsburg**	1995–1999	1995–2000	35–74	300,902	1560	60.4	286	0.14	49.6	171.8
**Barcelona**	1992–1995	1992–2000	35–79	893,601	1134	61.9	296	0.09	47.7	148.0
**Helsinki**	1993–1999	1993–2000	35+	297,410	4026	69.2	1301	0.45	30.1	173.4
**Rome**	1998–2000	1998–2001	35+	1,616,356	7384	67.0	1916	1.34	70.0	140.5
**Stockholm**	1994–1999	1994–2000	35+	548,113	7902	73.3	2856	1.23	22.8	81.2

**Figure 3:**
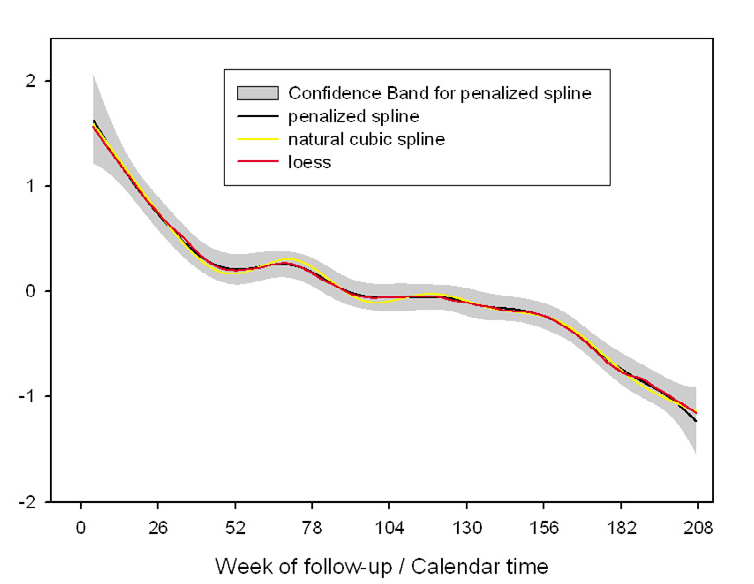
Exposure response function of the number of hospital readmissions occurring over time based on Possion regression analyses for Rome.

Figure 3 shows the smooth time-trend in the Poisson regression analyses using three different smoothing techniques: (a) penalized splines, (b) natural splines and (c) locally weighted least square smoothers (loess). Here the time-axis is the calendar-time axis and the functions shown in figure 3 correspond to the descriptive data in incidence rates in figure 1c. All functions suggest a decreased probability to observe hospitalizations due to any cardiac disease over time overlaid by seasonal variation. Thereby, they are able to capture the trend components discussed above remarkably well. The different smoothing approaches render quite comparable functions.

### The Effect of NO_2_

Table 2 compares the regression coefficients and standard errors for the different modeling approaches for a selected pollutant, (NO_2_) with the average between current and previous day concentrations. For the Poisson regression analyses sensitivity analyses are presented to assess the sensitivity of the results with respect to the confounder modeling. The selected models are shown in table 3. It is important to note that the same degrees of freedom were not required for exposure response functions in all cities, but that the number of cases observed determined the ability to model time-varying confounders.

**Table 2:** Comparison of the regression coefficients (beta) and standard errors (se) in the analyses of NO_2 _concentrations (average of current and previous day) and any cardiac readmission applying different statistical models and assessing the sensitivity of the results for confounder selection as listed in table 3.

	**Augsburg**		**Barcelona**		**Helsinki**		**Rome**		**Stockholm**		**pooled**		**Heterogeneity test**
**Poisson Regression Analyses**	beta	se	beta	se	beta	se	beta	se	beta	se	beta	se	p-value
Final Model	0.0119	0.0044	0.0068	0.0044	0.0015	0.0026	0.0041	0.0022	0.0013	0.0032	0.0039	0.0013	0.26
natural splines^a^	0.0119	0.0044	0.0066	0.0044	0.0012	0.0026	0.0037	0.0022	0.0012	0.0032	0.0036	0.0013	0.25
loess^b^	0.0119	0.0044	0.0065	0.0042	0.0015	0.0026	0.0040	0.0021	0.0014	0.0031	0.0038	0.0013	0.27
Removing Confounders	0.0117	0.0041	0.0078	0.0042	0.0019	0.0025	0.0049	0.0022	0.0011	0.0030	0.0044	0.0013	0.19
Adding Confounders	0.0122	0.0045	0.0051	0.0046	0.0024	0.0026	0.0043	0.0022	0.0018	0.0032	0.0041	0.0013	0.37
Apparent Temperature^c^	0.0115	0.0044	0.0067	0.0044	0.0012	0.0026	0.0039	0.0022	0.0013	0.0031	0.0037	0.0013	0.28
													
**Case-crossover Analyses**													
Stratified control selection	0.0144	0.0060	-0.0024	0.0065	0.0031	0.0031	0.0024	0.0026	0.0008	0.0036	0.0029	0.0016	0.32
													
**Cox-Extended Regression**													
Time-varying confounders	0.0123	0.0043	0.0046	0.0041	0.0013	0.0025	0.0034	0.0021	0.0017	0.0031	0.0035	0.0013	0.24
+ individual confounders	0.0123	0.0043	0.0047	0.0041	0.0011	0.0025	0.0034	0.0021	0.0016	0.0031	0.0034	0.0013	0.23

a) Model as described in table 3, p-splines replaced by natural splines

b) Model as described in table 3, p-splines replaced by loess

c) Final models described in table 3 with p-splines, apparent temperature replaces temperature

**Table 3:** Confounders included in the different models with the following abbreviations: S: penalized spline (* k = 30, otherwise k = 10), Poly: Polynomial with order in brackets, L: linear term, X: dummies, D: by design.

	**Trend**	**Temperature**	**Temperature difference**	**Relative humidity**	**Air pressure**	**Weekday**	**Holiday**	**Population decrease**
**Augsburg**								
Poisson Regression : Final	S	-	L	-	-	X	-	-
Poisson Regression : Remove	S	-	-	-	-	-	-	-
Poisson Regression : Add	S	S	L	L	-	X	-	-
Case-crossover	D	-	L	-	-	D	-	-
Extended Cox Regression	L	-	L	-	-	X	-	-
								
**Barcelona**								
Poisson Regression : Final	S	L	-	-	-	X	-	X
Poisson Regression : Remove	S	-	-	-	-	-	-	X
Poisson Regression : Add	S	L	L	-	-	X	-	X
Case-crossover	D	L	-	-	-	D	-	X
Extended Cox Regression	L	L	-	-	-	X	-	X
								
**Helsinki**								
Poisson Regression : Final	S	L	L	L	-	X	-	-
Poisson Regression : Remove	S	-	L	L	-	X	-	-
Poisson Regression : Add	S	S	S	L	-	X	-	-
Case-crossover	D	L	L	L	-	D	-	-
Extended Cox Regression	L	L	L	L	-	X	-	-
								
**Rome**								
Poisson Regression : Final	S*	S	-	L	-	X	X	X
Poisson Regression : Remove	S*	L	-	L	-	X	-	-
Poisson Regression : Add	S*	S	-	S	-	X	X	X
Case-crossover	D	S	-	L	-	D	X	X
Extended Cox Regression	L	Poly(2)	-	L	-	X	X	X
								
**Stockholm**								
Poisson Regression : Final	S	L	-	S	L	X	-	X
Poisson Regression : Remove	S	-	-	S	L	X	-	X
Poisson Regression : Add	S	L	L	S	L	X	-	X
Case-crossover	D	L	-	S	L	D	-	X
Extended Cox Regression	L	L	-	Poly(2)	L	X	-	X

**Figure 4:**
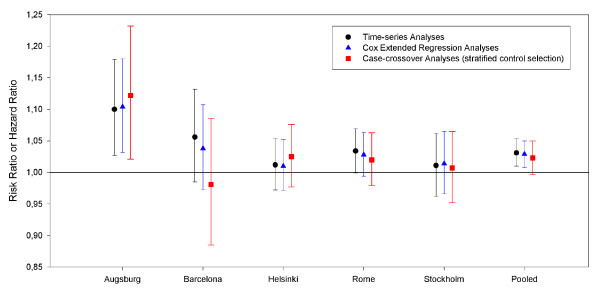
Effect estimates for the association between NO_2 _(8 μg/m^3^) hospital readmissions in MI survivors obtained in Poisson regression analyses and Extended Cox regression analyses of all five cities within the HEAPSS study and the pooled estimates based on a fixed effect model.

The different smoothing approaches show comparable air pollution effect estimates in the Poisson regression analyses. The pooled estimates for NO_2 _after confounder control by natural splines or loess functions are somewhat reduced, but one would still draw the same conclusions. Removing confounders overall increases the estimates whereas adding more confounders slightly decreases the regression coefficients. In the case-crossover analyses, the estimates are sometimes slightly higher (Augsburg) and sometimes slightly lower (Barcelona, Helsinki, and Rome) than the Poisson model estimates. The pooled estimate would suggest smaller regression coefficients and larger standard errors than Poisson regression analyses. The results of the extended Cox regression analyses were consistent with the results of the Poisson regression analyses in Augsburg, Stockholm and Helsinki. Slightly smaller effect estimates were obtained for Barcelona and Rome. Overall, the pooled estimates were slightly smaller than those obtained with Poisson regression analyses, but would also suggest an association between NO_2 _and hospital readmissions in MI survivors (figure 4). For extended Cox regression analyses individual characteristics were also considered as confounders in the analyses and the results were nearly identical to those obtained without consideration of individual characteristics. No strong evidence for heterogeneity of the city-specific effect estimates was observed and pooled random effect estimates were identical to the pooled fixed effect estimates (data not shown).

## Discussion

The major challenge of analyzing short-term health effects of air pollution in a cohort of diseased subjects is to consider simultaneously other time-varying confounders and the changes in the probability of a recurrent event due to the individual characteristics. In the case of myocardial infarction survivors, a major determinant of individual vulnerability is the time since index event due to the underlying healing of the heart tissue.

Three different approaches were considered in the analyses. The first one, Poisson regression analysis summarizes the events on a calendar time-axis. The data in this study demonstrate an example where the underlying probability of observing an event changes with the composition of the cohort. Therefore, time is not only a measure for slow trends and seasonal variation, but also represents the changing fractions of persons at high risk over time. Smoothing methods were used to attempt to model these three different components in the time trend. The recent discussion on smoothing techniques [11,12] was considered by the selection of three different approaches, namely natural splines, penalized splines and locally weight least square smoothers. All three methods gave quite comparable results and their function is consistent with a decreased risk of hospital readmission as the cohort ages and a seasonal variation in hospital readmissions. In the sensitivity analyses, results were robust to changes in the confounders selected in the final model.

The case-crossover method was chosen because it was designed to control for temporal changes by design. More specifically, the stratified referent period selection approach considered here controls for trends by design [19]. However, it has been noted that case-crossover analyses have reduced power compared to Poisson regression analyses [19]. In this study we observed slightly smaller effect estimates with larger standard errors. The case of a cohort with ongoing recruitment and dynamically changing composition has not been methodologically considered before. One may assume that the underlying changes in rates are constant within each stratum [21]. However, as observed in figure 1, panel C this assumption might be violated at the beginning of the study. Therefore, the changing number of subjects at risk might be responsible for the small differences observed between Poisson regression analyses, which explicitly consider the varying number of subjects at risk on a given day, and the case-crossover analyses. Nevertheless, case-crossover analyses were considered for these analyses because they quite elegantly allow analyses of subgroups.

Extended Cox regression analyses, on the other hand, were formulated to consider the present study design in the correct way. Here, the underlying risk is modeled by a hazard function *h*_0_(*t*) which is variable over time and considers both the changing hazard due to the recovery from the incident MI as well as changes in the composition of the cohort with respect to season and calendar year. The results are remarkably consistent with those obtained in the Poisson regression analyses. The practical disadvantage of the method is that the analyses are time-consuming, in particular if these models are used for the selection of time-varying confounders. Consideration of individual confounders did not change the association between NO_2 _and hospital readmissions. In contrast to a recently published similar approach, we chose time of follow-up instead of subject's age as time axis [22]. While these two approaches should give similar effect estimates we favor the model on the follow-up time axis as it more appropriately considers the changing risk levels within the cohort.

A recent review by the American Heart Association has highlighted the emerging evidence for the biological plausibility between ambient air pollution concentrations in urban areas and cardiovascular disease exacerbation [23]. However, effect estimates obtained from the general population might underestimate the risk of susceptible subpopulations, which have also higher baseline risks [24]. Cohort studies assessing the risk of susceptible populations are highly recommended to provide better estimates for risk assessment. For example, the age at first MI, socio-economic status as well as the time since the first MI might modify the risk of short-term air pollution exposures for an individual. All three methods might be used to assess the susceptibility of subgroups. The extended Cox regression analysis is the only method that would allow the estimation of the main effect of the considered effect modifier, but computation of the models is time-consuming.

Recent research indicated that spatial and temporal variability of long-term exposures to ambient particles may be important factors to consider [25,26]. Furthermore, future research might consider short-term fluctuations as well as individualized estimates of long-term exposures to ambient particles in assessing the health impact of environmental exposures. For these studies, Extended Cox regression analyses would be the method of choice. In its most simplistic version, one may estimate jointly the effect of homes exposed to high traffic together with air pollution concentrations from a central monitoring side. However, also more sophisticated approaches of exposure assessment building on spatio-temporal models such as for example described by Gulliver and Briggs [27] or Sahu and colleagues [28] can be foreseen.

## Conclusion

Of the three methods considered for the analyses of the HEAPSS Study, the Poisson regression approach and the extended Cox regression analyses gave similar results. Case-crossover analyses might underestimate the strength of the association in this specific setting, but the differences were small. Further methodological investigation may be warranted. From a practical point of view, Poisson regression analyses are less time-consuming, and therefore might be used for confounder selection and most of the analyses. However, replication of the results with Cox models is desirable to assure that the results are independent of the analytical approach used. For the identification of susceptible subgroups within a cohort of susceptible populations, case-crossover analyses might be the least time consuming approach, however, Extended Cox regression analyses would allow a joint estimation of the main effects and the effect modification.

## Competing interests

The authors declare that they do not have any competing interests. Fredrik Nyberg, employed by AstraZeneca, is also Lecturer in Epidemiology at Karolinska Institutet. Astra Zeneca did not contribute any direct financing to this study.

## Authors' contributions

AP wrote the first complete draft of the manuscript, has made substantial contributions to the design and the analysis, SvK conducted the analyses, made substantial contributions to the collection and analysis of the data and helped to draft the manuscript, NB made substantial contributions to design and data acquisition (Stockholm), AH made substantial contributions to data acquisition and validation (Augsburg), HL made substantial contributions to design and data acquisition (Augsburg), FN made substantial contributions to design and data acquisition (Stockholm), JP made substantial contributions to design and data acquisition (Helsinki); CAP made substantial contributions to data acquisition (Rome), MS made substantial contributions to data acquisition (Rome), JS made substantial contributions to design and data acquisition (Barcelona), PT made substantial contributions to design and statistical analyses, FF coordinated the project and conceived the design of the study. All authors contributed to the interpretation of the data, helped revising the manuscript, read and approved the final version of the manuscript.
